# Supplementary material for: Effect of Flank Rotation on the Photovoltaic Properties of Dithieno[2,3-d:2′,3′-d′]benzo[1,2-b:4,5-b′]dithiophene-Based Narrow Band Gap Copolymers
Source: Polymers (Basel). 2019 Feb 1;11(2):239. doi: 10.3390/polym11020239 (PMC6419082; doi:10.3390/polym11020239)
Supplement: Supplementary file 1 [file polymers-11-00239-s001.pdf]

polymers

MDPI

Supplementary Materials: Effect of Flanks Rotation on the Photovoltaic Properties of Dithieno[2,3-*d*:2′,3′-*d*′]benzo[1,2-*b*:4,5-*b*′]dithiophene-based Narrow Band Gap Copolymers

Mingjing Zhang, Liangjian Zhu, Pengzhi Guo, Xunchang Wang, Junfeng Tong, Xiaofang Zhang, Yongjian Jia, Renqiang Yang, Yangjun Xia and Chenglong Wang

1. Instruments and measurements

^{1}H NMR and ^{13}C NMR spectra were recorded on a Bruker DRX 500 spectrometer operating at 500 MHz and 125 MHz were referred to tetramethylsilane (TMS). Analytical gel permeation chromatography (GPC) was performed using a Waters GPC 2410 in THF relative to polystyrene standards. Thermal gravimetric analysis (TGA) was conducted on a TGA 2050 (TA instruments) thermal analyses system under a heating rate of 10 °C/min and a nitrogen flow rate of 20 mL/min. UV-Visible absorption spectra was measured on a UV-1800 spectrophotometer (Shimadzu. Co.). The X-ray diffraction (XRD) was carried out on a PANalytical X′Pert PRO diffractometer equipped with a rotating anode (Cu Kα radiation, λ = 1.54 Å). The cyclic voltammetry (CV) was measured on CHI600D electrochemical workstations (Shanghai Chenhua Co.) at a scan rate of 50 mV/s with a nitrogen-saturated solution of 0.1 M tetrabutylammonium hexafluorophosphate (Bu_4NPF_6) in acetonitrile (CH_3CN) with glass carbon and Ag/$AgNO_3$ electrode as the working and reference electrode, respectively. Tapping-mode atomic force microscopy (AFM) images were obtained on a NanoScope NS3A system (Digital Instrument). Transmission electron microscopy (TEM) images were acquired with a Tecnai G2 F20 (FEI, Hillsboro, OR, USA) transmission electron microscope at an accelerating voltage of 200 kV.

2. Preparation and characterization of the photovoltaic solar cells

A patterned indium tin oxide (ITO) coated glass with a sheet resistance of 10-15 Ω/square, was cleaned by a surfactant scrub, followed by a wet-cleaning process inside an ultrasonic bath, beginning with de-ionized water, followed by acetone and *iso*-propanol (*i*-PrOH). After oxygen plasma cleaning for 5 min, a 5 nm thick PFN layers were spin-casted onto the ITO. The active layers with a thickness ranging in the 100–110 nm, were then deposited on the top of the PFN-modified ITO by spin-casting from the chlorobenzene (CB) solution containing PDTBDT-TE-DTNT/$PC_{71}BM$, PDTBDT-T-DTNT/$PC_{71}BM$, with and without DIO as solvent additive. Then a 8 nm MoO_3 and 100

nm silver layer were evaporated with a shadow mask under vacuum of (1-5) × 10^{-5} Pa. The overlapping area between the cathode and anode defined a pixel size of device of 0.1 cm^2. The thickness of the active layers was determined by a Profile system (BRUKER VDS-9400 QS). The thickness of the evaporated cathode was monitored by a quartz crystal thickness/ratio monitor (SI-TM206, Shenyang Sciens Co.). Except for the deposition of the PFN layers, all the fabrication processes were carried on inside a controlled atmosphere in a nitrogen drybox (Etelux Co.) containing less than 1 ppm oxygen and moisture. The PCEs of the resulting polymer solar cells were measured under 1 sun, AM 1.5G (Air mass 1.5 global) condition using a solar simulator (XEC-300M2, San-EI Electric Co.) with irradiation of 100 mWois^{-2}. The current density-voltage (*J*-*V*) characteristics were recorded with a Keithley 2400 source-measurement unit. The spectral responses of the devices were measured with a commercial EQE/incident photon to charge carrier efficiency (IPCE) setup (7-SCSpecIII, Beijing 7-star Opt. In. Co.). A calibrated silicon detector was used to determine the absolute photosensitivity.

3. ^{1}H NMR and ^{13}C NMR Spectra

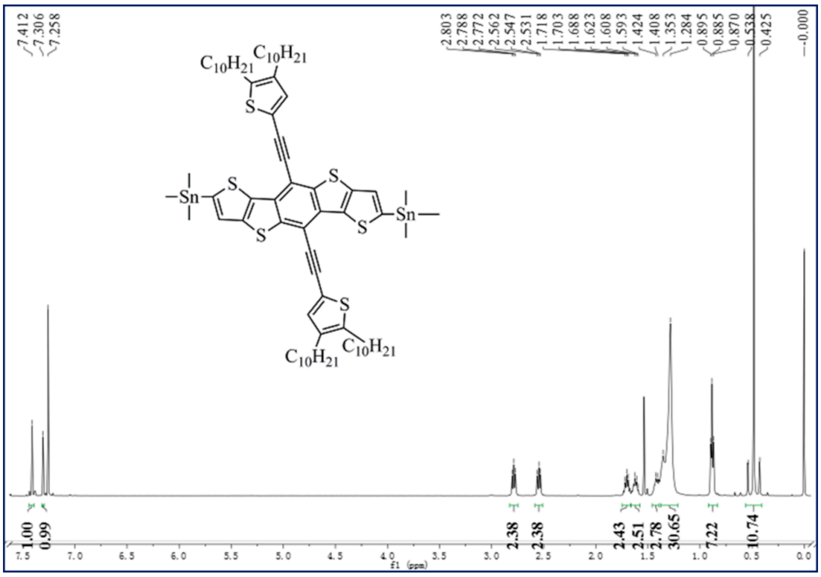

Figure S1. ^{1}H NMR spectrum of DTBDT-TESn in $CDCl_3$

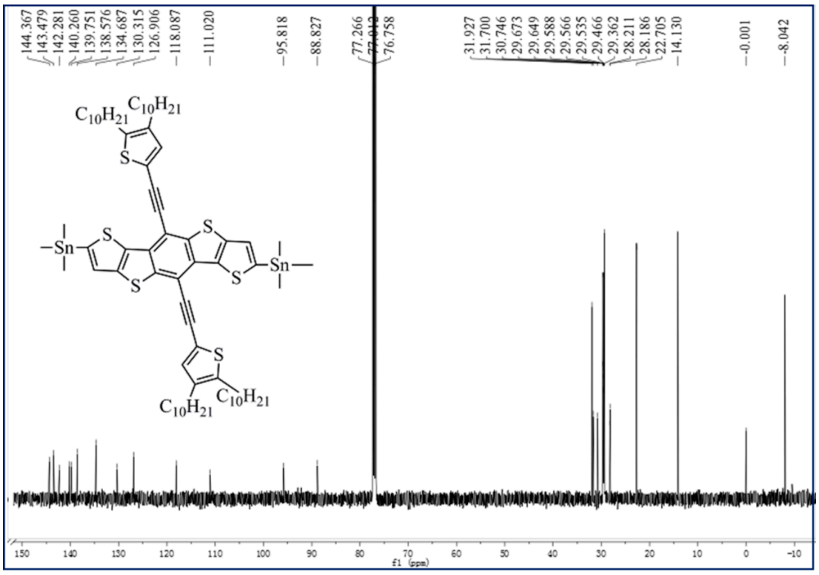

Figure S2. ^{13}C NMR spectrum of DTBDT-TESn in $CDCl_3$

4. TGA Plots

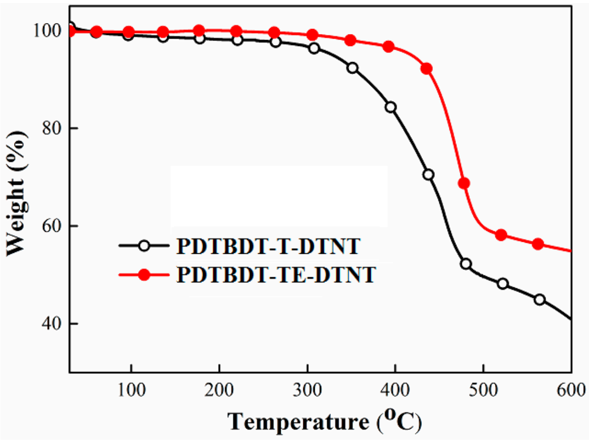

Figure S3. TGA plots of PDTBDT-TE-DTNT and PDTBDT-T-DTNT with a heating rate of 10 °C/min under an inert atmosphere.

5. Absorption spectra

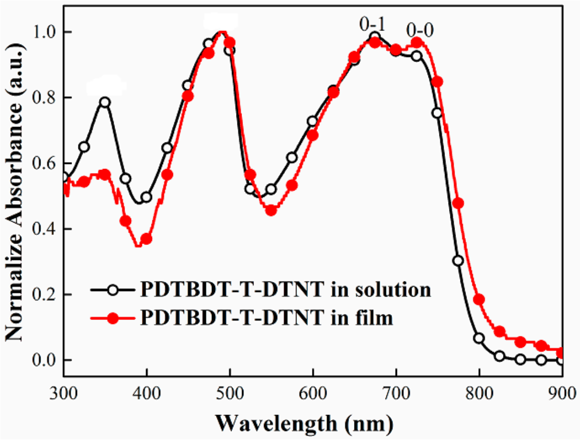

Figure S4. Normalized UV-Vis spectra of PDTBDT-T-DTNT in dilute chlorobenzene solution and solid thin film

6. Photovoltaic characteristics

Table S1. Parameters of *i*-PVCs from PDTBDT-TE-DTNT and $PC_{61}BM$ with different ratio

Active layer	Additives (DIO)	V_{OC} (V)	J_{SC} (mA/cm^2)	*FF* (%)	PCE (%)
**PDTBDT-TE-DTNT:$PC_{71}BM$ (W : W = 1:1)**	3%	0.64	8.67 (8.85) [a]	55.09	3.06
**PDTBDT-TE-DTNT:$PC_{71}BM$ (W : W = 1:2)**	3%	0.64	7.29 (7.02) [a]	45.58	2.27
**PDTBDT-TE-DTNT:$PC_{71}BM$ (W : W = 1:3)**	3%	0.64	5.84 (5.67) [a]	43.38	1.62

[a] The value in the parentheses is integrated current get from the IPCE testing system.

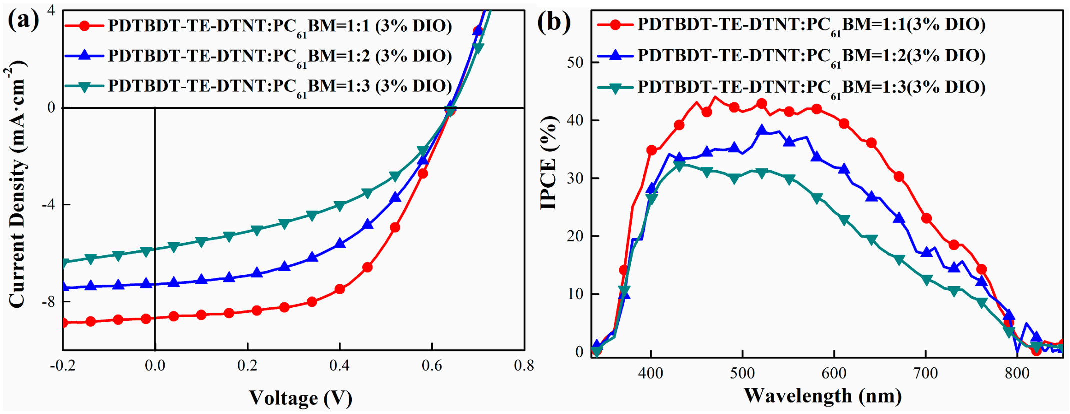

Figure S5. The *J-V* curves of PDTBDT-TE-DTNT with different weight rations to $PC_{61}BM$ (a) and the IPCE spectra (b) of corresponding *i*-PVCs.

7. Absorption spectra of blend films

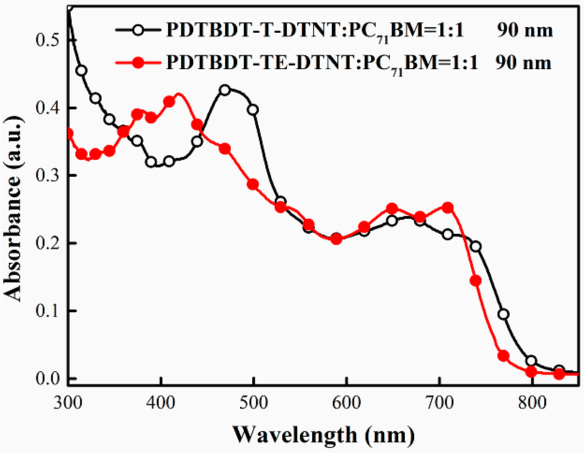

Figure S6. Absorption spectra of the copolymer/$PC_{71}BM$ blend films

8. Film morphology

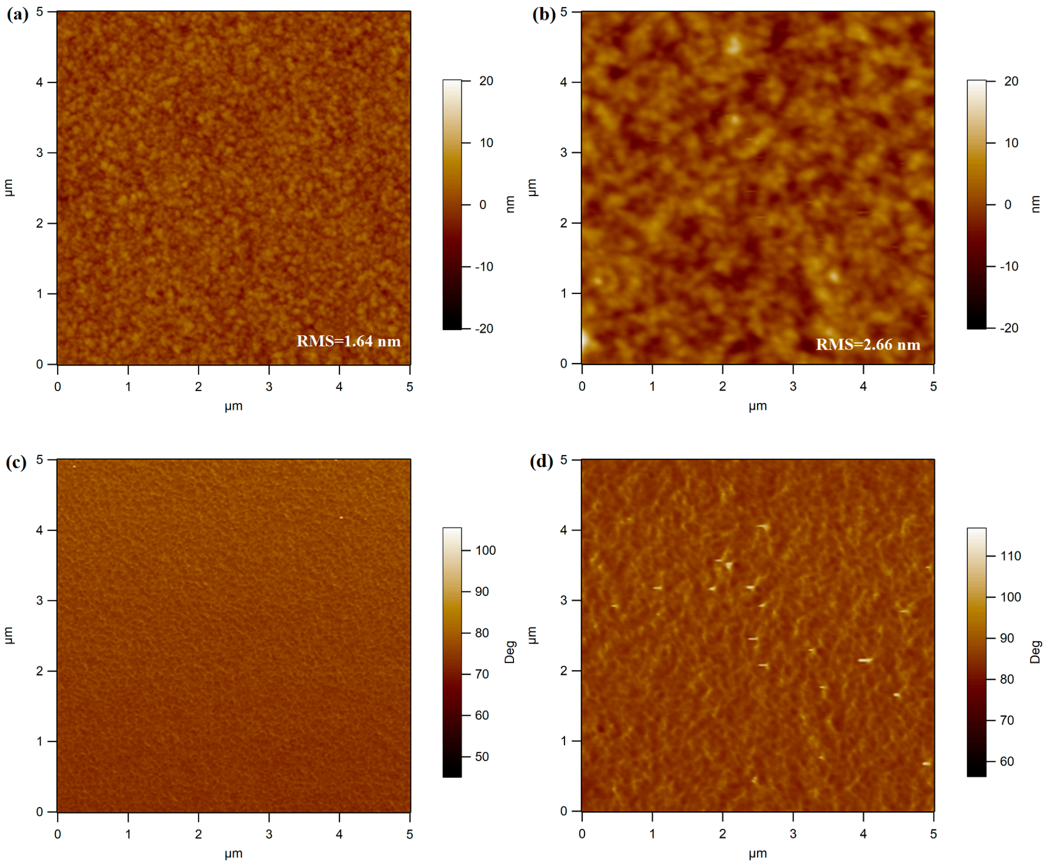

Figure S7. Tapping AFM height images (a, b) and phase image (c, d) (5 μm × 5 μm) for the blend films of PDTBDT-T-DTNT/$PC_{71}BM$ (1:1, 3% DIO, a, c) and PDTBDT-TE-DTNT/$PC_{71}BM$ (1:1, 3% DIO, b, d).

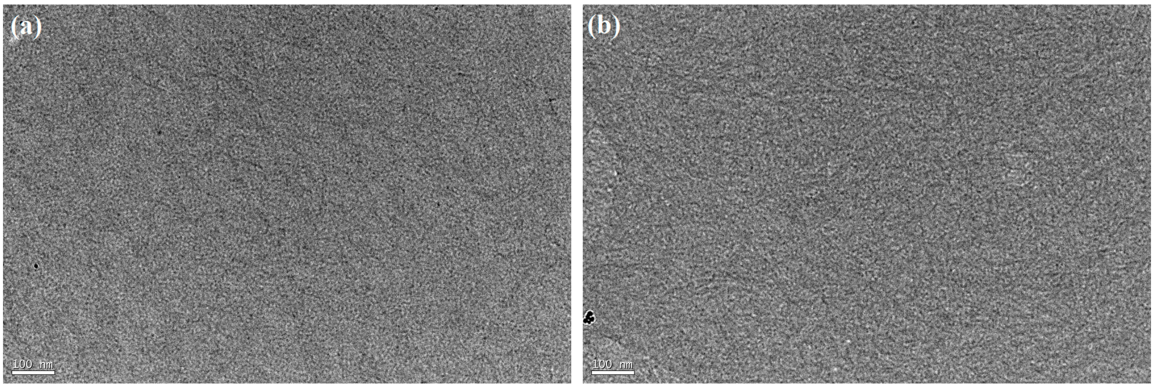

Figure S8. TEM topography images for the blend films of PDTBDT-T-DTNT/$PC_{71}BM$ (1:1, 3% DIO, a) and the blend films of PDTBDT-TE-DTNT/$PC_{71}BM$ (1:1, 3% DIO, b).
